# Adsorption of azide-functionalized thiol linkers on zinc oxide surfaces†

**DOI:** 10.1039/d0ra05127f

**Published:** 2021-01-29

**Authors:** Petia Atanasova, Maofeng Dou, Shravan R. Kousik, Joachim Bill, Maria Fyta

**Affiliations:** Institute for Materials Science, University of Stuttgart Heisenbergstr. 3 70569 Stuttgart Germany; Institute for Computational Physics, University of Stuttgart Allmandring 3 70569 Stuttgart Germany mfyta@icp.uni-stuttgart.de

## Abstract

A comprehensive understanding of the interactions between organic molecules and a metal oxide surface is essential for an efficient surface modification and the formation of organic–inorganic hybrids with technological applications ranging from heterogeneous catalysis and biomedical templates up to functional nanoporous matrices. In this work, first-principles calculations supported by experiments are used to provide the microstructural characteristics of (101̄0) surfaces of zinc oxide single crystals modified by azide terminated hydrocarbons, which graft on the oxide through a thiol group. On the computational side, we evaluate the specific interactions between the surface and the molecules with the chemical formula N_3_(CH_2_)_*n*_SH, with *n* = 1, 3, 6, 9. We demonstrate that the molecules chemisorb on the bridge site of ZnO(101̄0). Upon adsorption, the N_3_(CH_2_)_*n*_SH molecules break the neutral (Zn^*δ*+^–O^*δ*−^) dimers on ZnO(101̄0) resulting in a structural distortion of the ZnO(101̄0) substrate. The energy decomposition analysis revealed that such structure distortion favors the adsorption of the molecules on the surface leading to a strong correlation between the surface distortion energy and the interaction energy of the molecule. An azide-terminated thiol with three methylene groups in the hydrocarbon chain N_3_(CH_2_)_3_SH was synthesized, and the assembly of this linker on ZnO surfaces was confirmed through atomic force microscopy. The bonding to the inorganic surface was examined *via* X-ray photoelectron spectroscopy (XPS). Clear signatures of the organic components on the oxide substrates were observed underlying the successful realization of thiol-grafting on the metal oxide. Temperature-dependent and angle-resolved XPS were applied to examine the thermal stability and to determine the thickness of the grafted SAMs, respectively. We discuss the high potential of our hybrid materials in providing further functionalities towards heterocatalysis and medical applications.

## Abstract 2

Studying the interaction between organic molecules and metal oxide surfaces is key to the development and modification of organic–inorganic hybrids for application in heterogeneous catalysis, biomedical implants, and functional nanoporous matrices.

## Graphical abstract

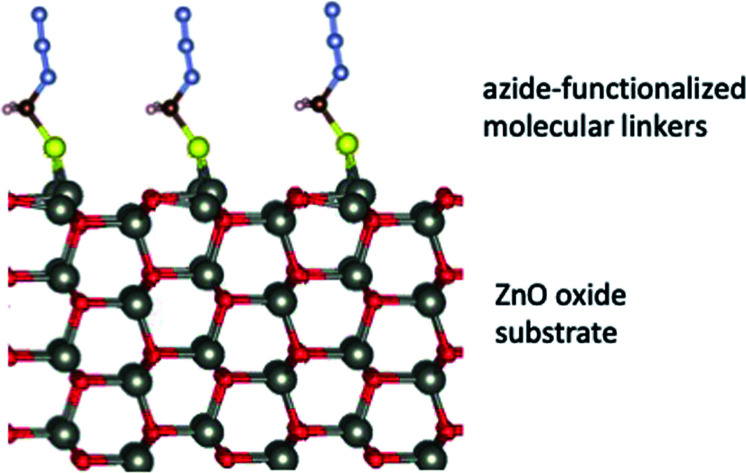

## 1 Introduction

Metal oxides, like AgO, Al_2_O_3_, ZrO_2_, ZnO, TiO_2_*etc.* are abundant in every day life and have been used for diverse applications. Their rigidity and resistance to stress are properties of high importance^1^ and have rendered them very good candidates for optoelectronics,^2^ as biomarkers,^3^ and pharmaceutical agents,^4^ in sensing,^5^ corrosion resistance applications,^6^ nanopatterning,^7^ controlled wetting,^8^*etc.* In order to realize these, though, the metal oxides are typically functionalized following common approaches for modifying the oxide surface properties.^5,9–11^ In general, the conjugation of (bio)organic molecules to inorganic surfaces can be achieved by applying different linking chemistries ranging from an electrostatic adsorption to a direct covalent bonding.^12^

Owing to its high carrier mobility, wide band-gap, large exciton binding energy and room-temperature luminescence,^13–15^ ZnO has been used extensively for photo and optoelectronic applications such as biomedical sensing, ultraviolet lasing and anode-materials for dye-sensitized solar cells.^16–19^ Furthermore, the high thermal and chemical stability of ZnO can be of potential significance in molecular heterogeneous catalysis, wherein these materials can be actively employed as porous solid-state catalytic supports. For the successful realization of the aforementioned applications, the ZnO surface needs to be functionalized with appropriate molecular linkers. Surface functionalization can impart enhanced electronic, photocatalytic and luminescence properties.^20–22^ In addition, the presence of organic molecules on the oxide surface can act as protective layer against corrosion.^23^ Specifically, ZnO (101̄0) due to its large specific surface area as in two-dimensional materials has a large affinity towards molecular adsorption. Accordingly, it could be used in sensing and catalysis applications for the adsorption of gas molecules, organic linkers, and reaction intermediates.^24–28^ An important approach that has been used to functionalize ZnO surfaces is the incorporation of covalently-bound self-assembled monolayers.^29,30^ These self-assembled monolayers are made up of well-ordered and strongly oriented functional organic molecules.^31^ In general, the core of a SAM typically consists of an alkyl chain with an anchor group that subsequently grafts onto the oxide surface *via* covalent bonding.^32^ The choice of the linker molecule is largely based on the application that is envisioned. A long-term goal is to provide molecular anchors to material surfaces for catalytic molecules. A specific application can be found in heterogeneous catalysis, in view of which the surface of ZnO can be modified with a bifunctional linker molecule possessing an anchor group and a terminal functional group. The terminal group offers a site for the immobilization of a catalytically active metal nanoparticle or coordination complex. For example, a linker molecule with an azide terminal group can conjugate with a catalyst with a terminal alkyne group *via* an azide–alkyne Huisgen cycloaddition reaction. These reactions are generally referred to as ‘Click’ reactions.^33,34^

For attachment to the ZnO surface, different anchor groups have been tested such as carboxyl, thiol,^35^ and phosphonate.^36^ Thiol groups are regarded as the most promising anchor groups to functionalize Au surfaces.^37^ Although other sulfur containing organic molecules, such as disulfides or alkanedithiols can be used as anchor groups for a SAM formation,^38,39^ the thiol group is known to lead to SAMs with the highest packing density.^40^ It has been shown that the functionalization of ZnO with thiol-based molecules improves the properties of ZnO-based materials and hence the function of the respective devices. For example, ZnO functionalization with dodecanethiol has shown to enhance the photoluminescence properties of ZnO films through a passivation of surface defects such as oxygen vacancies.^41^ The formation of a Zn–S bond on the surface of ZnO was shown to improve sensitivity for the detection of cardiac troponin-I.^42^ A thiol-based functionalization of ZnO has also led to thermally stable structures.^43^ A further inspection of the literature reveals studies on small molecular adsorption on zinc oxide surfaces. In many of these studies the adsorption takes place through a thiol group.^44^ These are mostly experimental and include among others the adsorption of hydrogen disulfide,^45^ alcohols^46^ or methanethiol^47^ on zinc and other similar oxides. In addition, other anchor types, such as phosphonic acids have also been successful in producing stable molecular layers on oxides.^48^ Nonetheless, despite the existing studies on thiol functionalized ZnO surfaces, the exact adsorption details regarding the linker attachment and the respective SAM formation are not fully understood due to the complexity of the surface structures and morphology of ZnO surfaces.

Most of the reports that describe the binding of self-assembled monolayers on ZnO only consider monofunctional linkers with long alkyl chains (>8 carbon atoms). There are very few studies that explain the molecular adsorption of bifunctional linkers with short alkyl chains on ZnO surfaces through experimental studies and theoretical models. Understanding the impact of the terminal azide-functionality on SAM formation and ordering is of great relevance to heterogeneous catalysis. These experiments have been designed in consonance with our larger research goals in which ZnO surfaces with azide-containing linkers shall be used as porous supports for the immobilization of molecular heterogeneous catalysts through ‘click’ chemistry. In this regard, we have modelled here the binding characteristics of N_3_(CH_2_)_*n*_SH (*n* = 1, 3, 6, and 9) molecules on a ZnO (101̄0). On the theoretical side, we focus on the energetic and electronic aspects of SAM adsorption on the substrate. Our experimental studies regarding the immobilization of ZnO (101̄0) surface with 3-azidopropyl thiol were performed to gain more insight into SAM film characteristics like bonding to the oxide surface, stability of the SAM layer at elevated temperature and film thickness. The aim of our work was to show the experimental realization of oxide surfaces modified in a way to provide an azide functionality. The theoretical part begins from that point and attempts to provide a more detailed insight into fundamental properties and aspects of such materials, by also varying the thickness of the SAM layer.

## 2 Methodology

### 2.1 Computational methods

The calculations are performed within the framework of density functional theory (DFT)^49,50^ as implemented in the Vienna *ab initio* simulation package (VASP).^51,52^ The basis set is described by the projector augmented wave (PWA) method.^53^ The exchange–correlation is described by generalized gradient approximation (GGA) functional of Perdew–Burke–Ernzerhof (PBE) functional.^54,55^ Dispersion corrections have been included through the DFT-D3 ansatz.^56^ The plane-wave basis set with a kinetic energy cutoff of 520 eV is used to expand the wave functions. The convergence thresholds for electronic and ionic relaxations are chosen to be 1.0 × 10^−5^ eV and 0.001 eV Å^−1^, respectively. In order to avoid spurious interactions of the adsorbed molecules with their periodic images, (3 × 3) supercells were considered for the surface. A large vacuum space of 30 Å was added in the direction perpendicular to surface to avoid interactions of the surface with atomic layers from its periodic images. The dipole correction was applied in the direction perpendicular to the surface. The Brillouin-zone integration was performed within the Monkhorst–Pack scheme using a 4 × 4 × 1 *k*-point mesh. We do not perform spin-polarized calculations, as for Zn – which is the atom involved in the bonding to the thiol end of the molecules – it has been shown that the relevant adsorption energies are not influenced by the polarization in the calculations.^57^

**Fig. 1:**
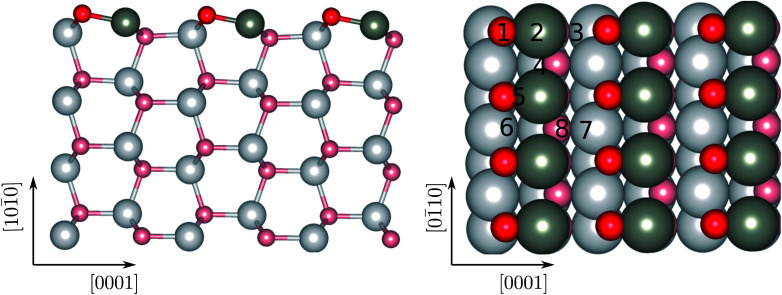
Side (left) and top (right) view of the free standing ZnO(1011̄0) surface after relaxation. The arrows denote the direction of the surfaces. The numbers correspond to the different adsorption sites investigated. The Zn and O atoms are given in dark gray, and red, respectively. The same color coding will be used throughout. On the right panel, the surface atoms are given in darker colors compared to the rest.

Using the approach above, we have modelled ZnO (101̄0) surfaces with four atomic layers as shown in Fig. 1. The wurtzite ZnO is a hexagonal crystalline phase, where each oxygen atom is tetrahedrally coordinated to four Zn atoms and *vice versa*. High-resolution scanning tunneling microscopy measurements have suggested that the low Miller index planes, namely (101̄0), (112̄0), (0001), and (112̄1), are most stable surfaces of wurtzite ZnO.^58^ Among them, the (101̄0) surface is the energetically most favorable and dominates in nanoscale structures, such as nanorods and nanoparticles.^59^ We therefore choose the (101̄0) surface of ZnO as a model ZnO surface in order to study the adsorption of azide-functionalized thiol molecules. The ZnO(101̄0) is a non-polar surface. For this surface, we have used the lattice constants previously obtained by similar theoretical studies.^60^ According to these, the ratio *c*/*a* of the lattice constants is 1.61. After relaxation, the two-coordinated oxygen atoms shift upwards towards the vacuum and the two-coordinated zinc atoms shift downwards as seen in Fig. 1. The bond length of the surface Zn–O decreases to 1.87 Å compared to that in bulk phase, *e.g.*, 2.02 Å. As a result, neutral (Zn^*δ*+^–O^*δ*−^; *δ* the partial charge) dimers form and stabilize the surface.

**Fig. 2:**
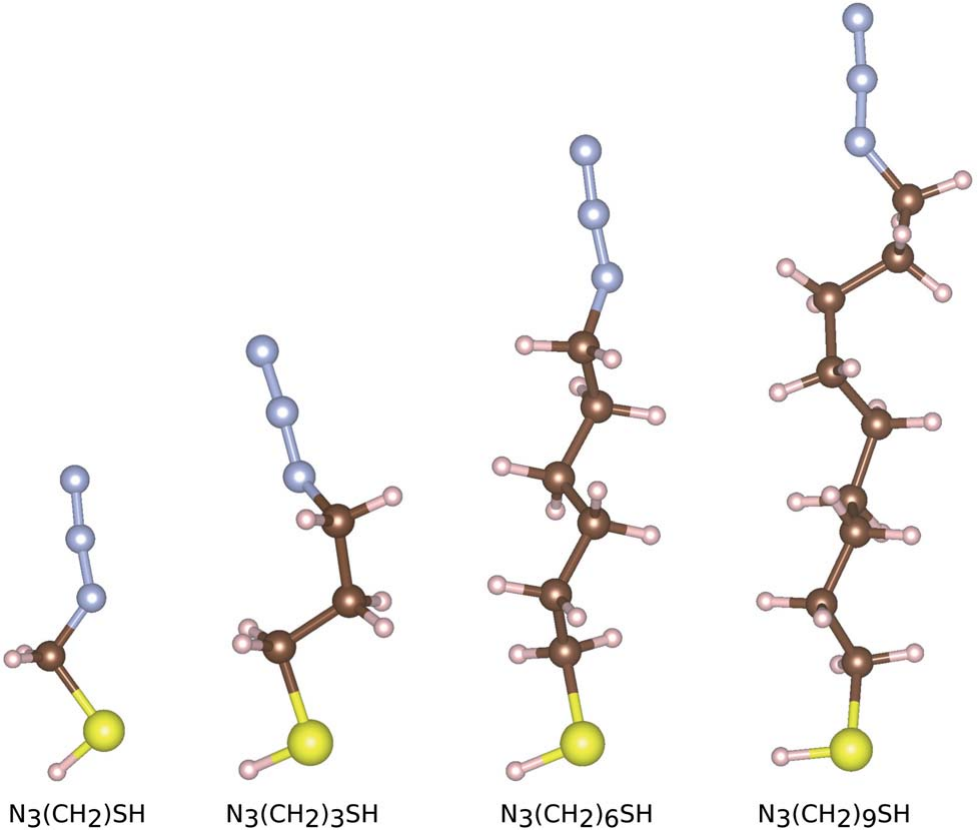
The relaxed free standing configurations of thiol functionalized linkers N_3_(CH_2_)_*n*_SH with different alkyl-chain lengths *n*. The S, H, C, and N, atoms are given in yellow, white, brown, and light blue, respectively. The same color coding will be used throughout.

In order to study the adsorption of small linker molecules on the ZnO(101̄0), here we consider molecular linkers made of short alkyl-chains functionalized on one end (the head) with a thiol group and on the other terminal with an azide group. These molecules are denoted as N_3_(CH_2_)_*n*_SH, with *n* = 1, 3, 6, 9 showing the length of the alkyl chain. The thiol group is the one that grafts on the zinc oxide surface, while the azide group provides further functionalization possibilities of the templates. Four thiol molecules of different lengths are separately relaxed as shown in Fig. 2. As references, we use the cases of the free standing (isolated and *in vacuo*) molecules and surfaces. For both components, the surface and the molecule, a pre-relaxation took place prior to attaching the molecule on the surface. In the case of the surface, all four atomic layers were pre-relaxed, while for the molecules, all atoms were allowed to pre-equilibrate. For both the surface and the molecules, the structure and configurations, respectively from the separate pre-equilibration were used for the next step related to the adsorption. Once the components are brought together, *i.e.* a molecule is attached on the surface, all atoms, but the bottom two layers of the surface are allowed to structurally equilibrate. The same pre-equilibrated zinc oxide surface was used for all subsequent calculations with the adsorbed molecules. After the molecules have been pre-relaxed, we remove the hydrogen atom of their thiol group in order to dock them on the zinc oxide surface. In order to distinguish between the two cases for the molecules, we use the notation N_3_(CH_2_)_*n*_S^−^ for the free standing molecules (including the hydrogen atom in the thiol group) and N_3_(CH_2_)_*n*_S^−^ (with only the sulfur in the thiol group) for the molecules being adsorbed on the zinc oxide surface.

For optimizing the adsorption, we perform an adsorption site search, as will be discussed next, during which the sulfur of each molecule is adsorbed on the surface. In this way thin SAMs of N_3_(CH_2_)_*n*_S^−^ are formed on ZnO(101̄0). Note again, that for the adsorption the passivating hydrogen of the thiol is removed. In order to assess the stability of the functionalized zinc oxide surface, we evaluate the adsorption energy (*E*_ads_) of the molecule on the ZnO(101̄0). Considering that the surface functionalization takes place in a solvent solution, it can be assumed that the adsorption process is a dissociative adsorption. During this, the H atom is first dissociated from the thiol group. At a second step, the dissociated H cation is adsorbed on the O atom on the surface. Then the dissociated H cation interacts with the solvent molecules forming a cation complex. In the experiments, the solvent is an ionic liquid and should strongly interact with the H cation. Accordingly, this interaction is expected to be stronger than that of the H cation with the surface oxygen. Due to the complexity of the solvent and its interaction with the H cations, based on the above considerations, we do not take into account the H cation in the calculation of the adsorption energy, which is defined though we approximately calculated the adsorption energy through

1 *E*_ads_ = *E*_ZnO+mol_ − *E*_ZnO_ − *E*_mol_,

where *E*_ZnO+mol_ is the total energy of the ZnO(101̄0)-functionalized surface, *E*_ZnO_ is the total energy of the clean free standing surface, and *E*_mol_ is the total energy of the free standing N_3_(CH_2_)_*n*_S^−^ in vacuum. Using the energy of molecular H_2_ as the chemical potential for H, leads to positive adsorption energies, and is thus not used in the equation. In order to analyze the characteristics of the molecular adsorption on the surface, we will perform an energy decomposition. Upon adsorption, the geometry of the molecule and the surface are distorted compared to their free standing counterparts. In energy terms, this distortion can be evaluated through the calculation of the energy of the distorted molecule and that of the distorted ZnO(101̄0). The distortion energy (*E*^ZnO^_dis_) of the substrate is evaluated through:^61^

2 *E*^ZnO^_dis_ = *E*_ZnO′_ − *E*_ZnO_,

where *E*_ZnO′_ is the total energy of the ZnO(101̄0) surface with the distorted structure due to the molecular adsorption and *E*_ZnO_ is the total energy of the free standing ZnO(101̄0). In an analogous manner, the distortion energy of the molecule (*E*^mol^_dis_) can be given through

3 *E*^mol^_dis_ = *E*_mol′_ − *E*_mol_,

where *E*_mol′_ is the total energy of the adsorbed molecule, which has been distorted due to adsorption and *E*_mol′_ is the total energy of the free standing molecule. The interaction energy (*E*_int_) of the molecule and the substrate can thus be calculated through the binding energy between the distorted molecule and the distorted ZnO(101̄0) substrate through

4 *E*_int_ = *E*_ZnO+mol_ − *E*_ZnO′_ − *E*_mol′_

Accordingly, the adsorption energy can be decomposed into the interaction energy between the ZnO(101̄0) and the molecule and the distortion energy of the adsorbate molecule and the ZnO(101̄0), through *E*_ads_ = *E*_int_ + *E*^ZnO^_dis_ + *E*^mol^_dis_.

In order to promote a qualitative comparison to our experimental results, we have also calculated the core electron binding energy (BE). This is calculated using the initial state approximation, which is based on a full self-consistent calculation including the frozen core electrons.^62–64^ The PAW approach was used to treat the core electrons. The BE is referred to the Fermi energy (*E*_F_). The BE shift, ΔBE, is determined through ΔBE = BE − BE_ref_. In this equation, BE_ref_ is the BE of the N_3_(CH_2_)_3_S in vacuum or that of the ZnO(101̄0) slab, respectively. The calculated BE is not expected to quantitatively match to the experimental BE, as the experimental and theoretical structures are not identical. However, it should allow for a comparison of trends between our methods.

### 2.2 Experimental methods

#### 2.2.1 Materials

*S*-(3-Azidopropyl)thioacetate (CH_3_COS(CH_2_)_3_N_3_, 95%) was purchased from Sigma Aldrich and used without further purification. Polished ZnO substrates with surface orientation (101̄0) and with the dimensions 10 × 10 × 0.5 mm^3^ were purchased from Crystal GmbH (Germany). Ethanol (for spectroscopy, Uvasol, water content < 0.05%) used for the SAM formation was purchased from Merck KGaA.

#### 2.2.2 Synthesis of 3-azidopropyl thiol

*S*-(3-Azidopropyl)thioacetate (1 g, 6.28 mmol) was dissolved in 10 mL ethanol in a three neck, round bottom flask under inert atmosphere. NaOH solution (516.16 mg in 1.85 mL degassed water, 19.9 mmol) was added drop-wise under stirring. The reaction mixture was refluxed for 3 hours and subsequently it was chilled to room temperature. The reaction solution was neutralized with 4.3 mL degassed 2 M HCl solution. Then, it was transferred in a separatory funnel under inert gas, and the organic phase was isolated through extraction with degassed diethyl ether. The organic layer was washed with 10 mL degassed water and the solvent was removed under vacuum. ^1^H̲ NMR (300 MHz, CHCl_3_-d, *δ* ppm): *S*-(3-azidopropyl)thioacetate: 1.87 (quin, 2H), 2.35 (s, 3H), 2.95 (t, 2H), 3.37 (t, 2H), 3-azidopropyl thiol 1.99 (quin, 2H), 2.76 (t, 2H), 3.44 (t, 2H). FT-IR (cm^−1^): *S*-(3-azidopropyl)thioacetate: 2928, 2918 (C–H stretching), 2090 (–N_3_ stretching), 1685 (C=O stretching), 1446, 1353, 1276, 1246, 1131, 1108, 950, 622, 558. 3-Azidopropyl thiol: 2926, 2917 (C–H stretching), 2090 (–N_3_ stretching), 1447, 1342, 1237, 1153, 1027, 976, 911, 860, 770, 631, 553.

#### 2.2.3 Self-assembly of 3-azidopropyl thiol

Prior to use, the ZnO substrates were cleaned with ethanol, treated with O_2_-plasma (10 min, 30 W), washed with ethanol again and dried under nitrogen stream. The cleaned substrates were placed in the thiol solution (20 mM in ethanol) under inert atmosphere for 24 hours. The SAM-functionalized substrates were rinsed thoroughly with ethanol and blown dry with nitrogen. Note that overall, we have carried out multiple measurements and have confirmed the repeatability of our experiments.

#### 2.2.4 Experimental analysis

Atomic force microscopy (AFM) was used to image the bare and SAM-functionalized ZnO substrates. The images were recorded on a Digital Instruments MultiMode 8 from Bruker with a NanoScope 5 controller operated in tapping mode. Silicon cantilevers and PPP-NCHR-W (Nanosensors) n^+^ doped tips with resistivity 0.01–0.02 Ω cm were used. ^1^H-NMR measurements were conducted on Bruker Avance 500 NMR spectrometer operating at 500 MHz field. Spectral processing and analysis were done using an CD/Spectrus Processor. Fourier transform infrared spectroscopy (FT-IR) analysis of the samples was performed on a Bruker FT-IR spectrometer. The samples were investigated in the range: 500–4000 cm^−1^ and the spectra were analyzed by the ACD/Spectrus Processor. Angle-resolved (AR) and temperature-dependent X-ray photoelectron spectroscopy (XPS) measurements were performed using Thermo Scientific™ Theta Probe Angle-Resolved X-ray Photoelectron Spectrometer System (Theta Probe ARXPS). A nonmonochromatic Twin gun X-ray source equipped with Al-Kα radiation (1486.6 eV) with an operating power of 100 W was used to stimulate a photoemission. The XPS spectra were taken with a pass energy of 200 eV (survey scan, 1 eV step, dwell time 50 ms) and several pass energies (single peaks, snap scan mode with number of frames between 400 and 700, 1s time for each frame). The binding energies were calibrated relative to the C 1s binding energy set up at 284.8 eV. For temperature-dependent measurements, the samples were first analyzed at room temperature. The samples were then transferred in UHV to a connected sputter chamber fabricated by CreaTec Fischer & Co GmbH and heated at a certain temperature (100 °C, 300 °C) for 30 min in UHV. The samples were allowed to cool down to room temperature and the spectra were recorded. ARXPS spectra were used to calculate the SAM film thickness from the attenuation of the Zn 2p_3/2_ photoelectron line. This was done by measuring the intensity of the Zn 2p_3/2_ peak (*I*_Zn_) at different angles (*θ*). The thickness of the SAM film can then be calculated by parameterizing (*I*_Zn_) as a function of the cosine of the angle (*θ*) using the equation *I*_Zn_ = *I*^∞^_Zn_ exp(−*t*/*λ*_Zn_ cos *θ*),^65^ where ‘*t*’ is the film thickness and *λ*_Zn_ is the electron inelastic mean free path of ZnO. The *λ*_Zn_ value is dependent on kinetic energy and is required to determine the thickness of the SAM film. The *λ*_Zn_ value for electrons with the kinetic energy of the Zn 2p_3/2_ peak was calculated using the TPP-2M equation^66^ from the NIST inelastic mean free path database.^67^ For our calculations, a *λ*_Zn_ value of 11.90 Å corresponding to the kinetic energy (487 eV) of the Zn 2p_3/2_ photoelectron line was used.

## 3 Results and discussion

### 3.1 Adsorption sites on ZnO(101̄0)

**Fig. 3:**
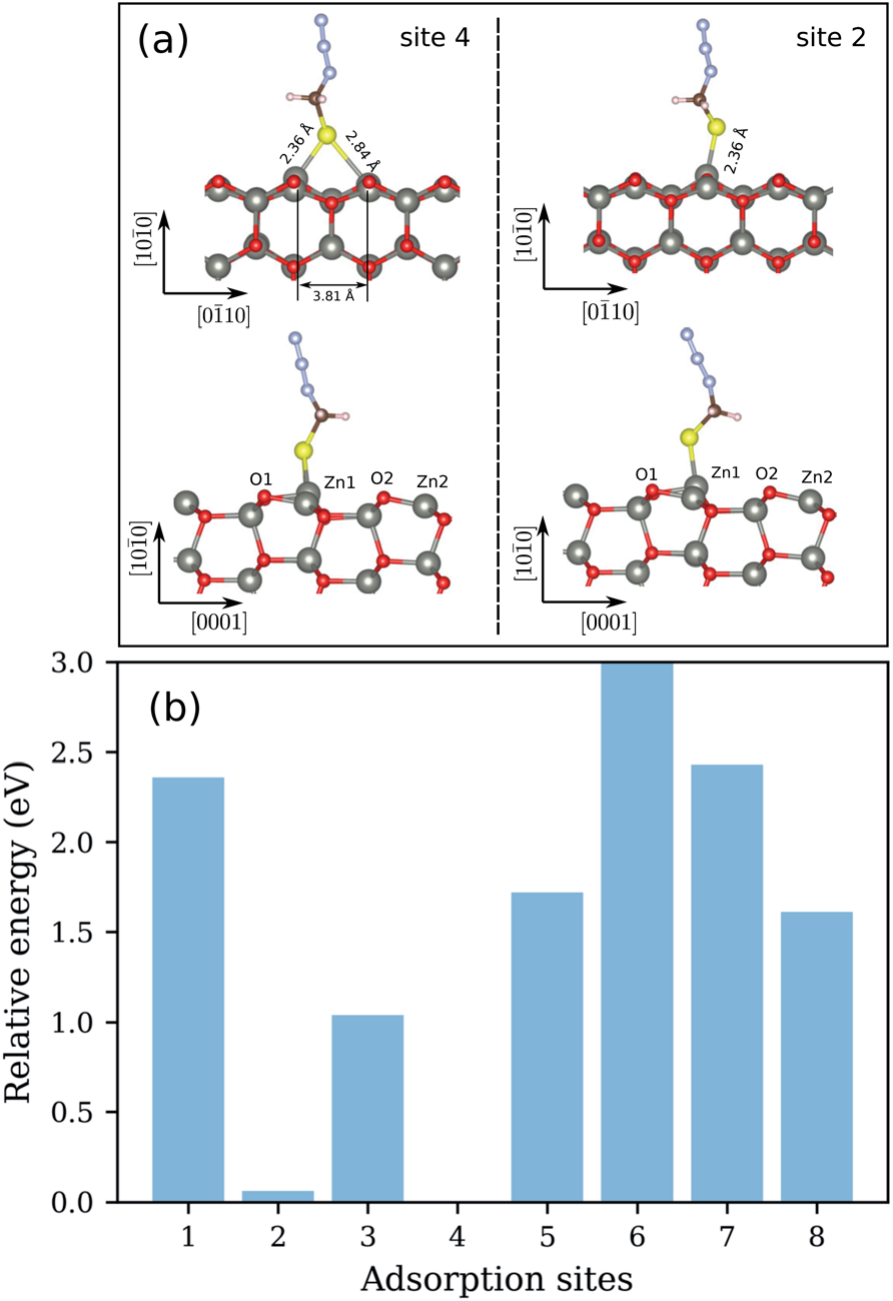
(a) Side views of the relaxed configurations of N_3_CH_2_S^−^ adsorbed on ZnO(101̄0) with the lowest (site-4) and second lowest (site-2) adsorption energies. The bond distances of the molecule to the surface are also shown. (b) The calculated total relative energies of N_3_CH_2_S^−^ adsorbed on different sites of the surface of ZnO(101̄0).

We begin the discussion with the analysis of the computational results and the preferential adsorption sites of the molecules on the surface. The possible adsorption sites are generated using the Catalysis Kit (CatKit).^68^ A symmetry analysis is applied to reduce to repeating adsorption sites. The eight possible (high-symmetry) adsorption sites on ZnO(101̄0) are enumerated in Fig. 1. The site-search was performed for N_3_CH_2_S^−^ and the findings were adopted for the longer molecules. We have compared eight adsorption sites and no stable adsorption was found when the molecule was positioned on a top site of an oxygen atom of the surface. The adsorption site with the most favorable energy is the bridge adsorption (site-4) in which the N_3_CH_2_S^−^ binds to the two neighboring surface Zn atoms *via* two Zn–S bonds with the Zn–S bond-length of 2.36 and 2.84 Å respectively as depicted in Fig. 3(a). The corresponding adsorption energy is −0.73 eV which is within the range of chemisorption energies. The second lowest energy (−0.68 eV) corresponds to the on-top adsorption (site-2) in which the N_3_CH_2_S^−^ binds to the surface Zn atom *via* a Zn–S bond, with the Zn–S bond-length being stabilized at 2.31 Å again as shown in Fig. 3(a). The bond lengths are comparable to that of ZnS single crystal (2.36 Å). Note that, similar trends on molecular adsorption on similar sites on the oxide surface have been reported also for other molecules. For example, an ethanethiol molecule also prefers to form two bonds with two of the neighboring zinc atoms on the surface.^69^

The energy difference between the two most stable configurations is very small, thus both are expected to occur in the SAM formation on the zinc oxide surface. The other structures are significantly higher in energy and cannot be expected to be practically achievable. The calculated total energies of N_3_CH_2_S^−^ adsorbed on different sites of the ZnO(101̄0) are shown in Fig. 3(b). All total energies are shown relative to the total energy of the most stable configuration corresponding to the adsorption of N_3_CH_2_S^−^ on the most favorable site-4 of the surface. For a better understanding of the adsorption, recall that the ZnO(101̄0) surface is a non-polar surface in which the Zn–O dimers stabilize the surface. An adsorption of N_3_CH_2_S^−^ breaks this surface dimer and gives rise to a structural distortion on the ZnO surface. When N_3_CH_2_S^−^ binds to the surface Zn atom, the Zn atom rises outwards and the neighboring surface O atom moves inwards whereas the rest remain almost fixed. As a result, the bond length of Zn–O, which binds to the N_3_CH_2_S^−^ is increased to 1.96 and 1.93 Å (Table 1) for site-2 and site-4 adsorption, respectively. The Zn–O bond lengths of the rest Zn–O pairs on the surface remain at 1.87 Å as in the clean surface. Overall, the surface structure is only deformed locally for the configurations of both site-2 and site-4 adsorption. The bonding distances at the surface after geometry relaxation are summarized in Table 1.

**Table 1:** Optimized bond length *d* (in Å) for the N_3_CH_2_S^−^ adsorption on ZnO(101̄0). The atomic labels refer to Fig. 3

Parameters	Site-2	Site-4
*d*_Zn–S_	2.31	2.36; 2.84
*d*_Zn–O1_	1.96	1.93
*d*_Zn–O2_	1.87	1.87
*d*_Zn–O_	1.87	1.87
*d*_Zn–O1_/*d*_Zn–O_	1.05	1.03
*d*_Zn–O2_/*d*_Zn–O_	1.00	1.00

**Fig. 4:**
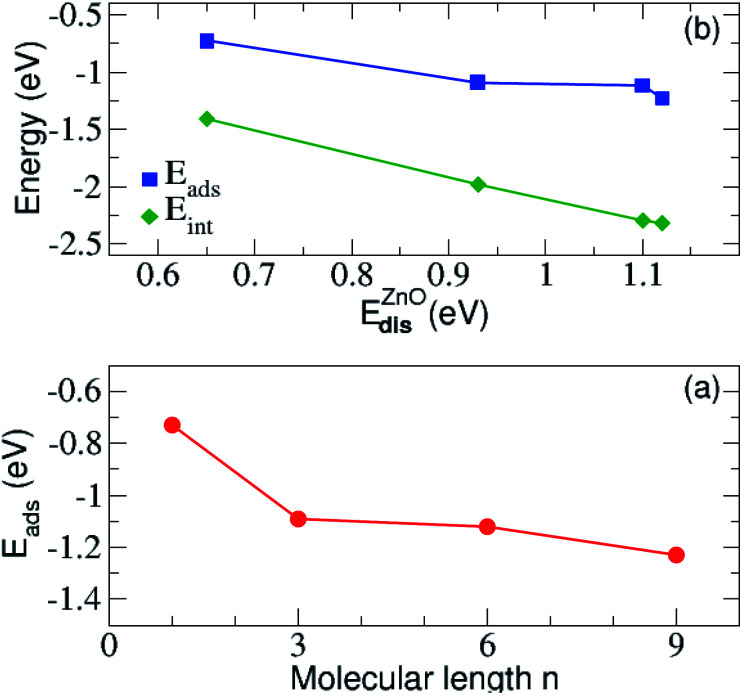
(a) The adsorption and the interaction energy between the N_3_(CH_2_)_*n*_S^−^ molecule and ZnO(101̄0) surface as a function of the ZnO(101̄0) distortion energy. In (b) the variation of the adsorption energy with respect to the alkyl length *n* are shown.

We have next assessed the energetic components of the molecular adsorption and the formation of the SAMs on the zinc oxide surface, by taking into account the most favorable adsorption on site-4 of the surface. All energies are summarized in Table 2. It is evident from the data in this table that the adsorption becomes stronger with the increase of the alkyl chain length *n*. Similarly, the interaction energy between ZnO(101̄0) and N_3_(CH_2_)_*n*_S^−^ lowers with increasing the length of the carbon chain of N_3_(CH_2_)_*n*_S^−^. The adsorption energy is plotted against the energy distortion of the surface and the alkyl length in Fig. 4 and shows a drop in both cases. Accordingly, the adsorption becomes stronger for the adsorption of longer molecules and for larger distortion energies. These two results indicate that longer molecules lead to a larger surface rearrangement, that is distortion, in order to accommodate the molecule. The distortion energy of ZnO(101̄0) follows the same trend as the interaction energy. The largest distortion cost for the adsorption corresponds to the strongest interaction between ZnO(101̄0) and the molecule. This suggests that though the surface is being distorted, the respective structural changes favor the interaction between the surface and the molecule. The distortion energies for all molecules also given in Table 2 and are all very small denoting that the molecules are not considerably distorted in order to bond and accommodate on the surface. We observe a small increase with the molecule length. This behavior is probably related to the fact that the longer the molecule the more atoms need to slightly rearrange due to the bonding to the surface. This is observed until length *n* = 9 for which the distortion energy decreases again. A simple physically intuitive argument based also on our experimental insight (not included here) is that above this length, the molecules are too stiff, so that only their lower atoms are distorted due to adsorption. Note also, that though the differences in distortion and interaction energies as the length *n* changes from *n* = 1 to *n* = 3 and from *n* = 6 to *n* = 9 is relatively similar (see Table 2) this trend is not reflected in the adsorption energy. The adsorption energy rather reflects the variation of the molecular distortion, which seems to slowly saturate with increasing chain length, as mentioned above.

**Table 2:** The different energies for the relaxed geometries of N_3_(CH_2_)_*n*_S^−^ (*n* = 1, 3, 6, 9) adsorbed on the lowest energy site-4 of ZnO(101̄0). The respective Badder charge differences of the molecule N_3_(CH_2_)_*n*_S^−^ (Δ*Q*_mol_) and that of ZnO(101̄0) (Δ*Q*_ZnO_) are also given

Parameters	*n* = 1	*n* = 3	*n* = 6	*n* = 9
*E*_ads_	−0.73	−1.09	−1.12	−1.23
*E* ^mol^_dis_	0.004	0.053	0.071	0.055
*E* ^ZnO^_dis_	0.65	0.93	1.10	1.12
*E*_int_	−1.41	−1.98	−2.30	−2.32
Δ*Q*_mol_	−0.18	−0.23	−0.23	−0.23
Δ*Q*_ZnO_	0.18	0.23	0.23	0.23

In addition, the distortion energy of N_3_(CH_2_)_*n*_S^−^ compared to the free standing N_3_(CH_2_)_*n*_S^−^ is very small, indicating a negligible effect of adsorption on the distortion of the molecule. In addition, we find a linear correlation of the distortion energy of the substrate and its interaction energy with the molecule as observed in Fig. 4(b). Accordingly, the highly distorted ZnO(101̄0), with corresponding large distortion energies, strongly interacts with the adsorbed molecule. More importantly, the presence of the molecule close to the surface and the corresponding bonding formation with the latter distorts the surface in a way to control the adsorption process, thus its strength. This is an important finding in view of designing materials, as according to the relation in the figure, the distortion energy could be an effective feature used in Machine Learning in order to predict materials with desired adsorption properties.^70^ Moreover, a similar correlation to the one in Fig. 4(a) was also observed in benzene adsorbed on a Pt surface.^61^ In that case, the distortion of the benzene molecule gives rise to a reduced gap between the lowest occupied and highest unoccupied state, thus stronger interactions.

As a final indication from our computational modeling, we turn to representative electronic features of the thin SAMs on zinc oxide. For this, we have performed a Bader charge analysis.^71^ The calculated net charge (Δ*Q*_ads_) of the shortest (N_3_CH_2_S^−^) molecule adsorbed on the surface is −0.18 |e|. This indicates a movement of charge from the ZnO substrate towards the molecule, leading to a partially charged N_3_CH_2_S^*δ*−^. The charge analysis on each atom further reveals that the negative charges in N_3_CH_2_S^*δ*−^ primarily originate from the first layer of the ZnO surface. We provide the charge differences in Table 2. These differences correspond to the differences of the Bader charge between the free standing and the adsorbed cases, respectively. Specifically, Δ*Q*_mol_ corresponds to the change in the Bader charge between the free standing and adsorbed molecule, while Δ*Q*_ZnO_ is the change between the free standing and modified surface. It is evident from this table, that in all cases part of the surface charge re-accommodates on the molecule. Accordingly, the adsorbed molecule attracts electrons from the substrate. The amount of charge that is being extracted from the zinc oxide surface and is being localized on the molecule is similar for all molecular lengths investigated here, apart from the shortest molecule. At least up to the lengths we have studied here, this trend indicates that the charge the surface loses is not dependent on the molecular length, but on the nature of the anchor group.

**Fig. 5:**
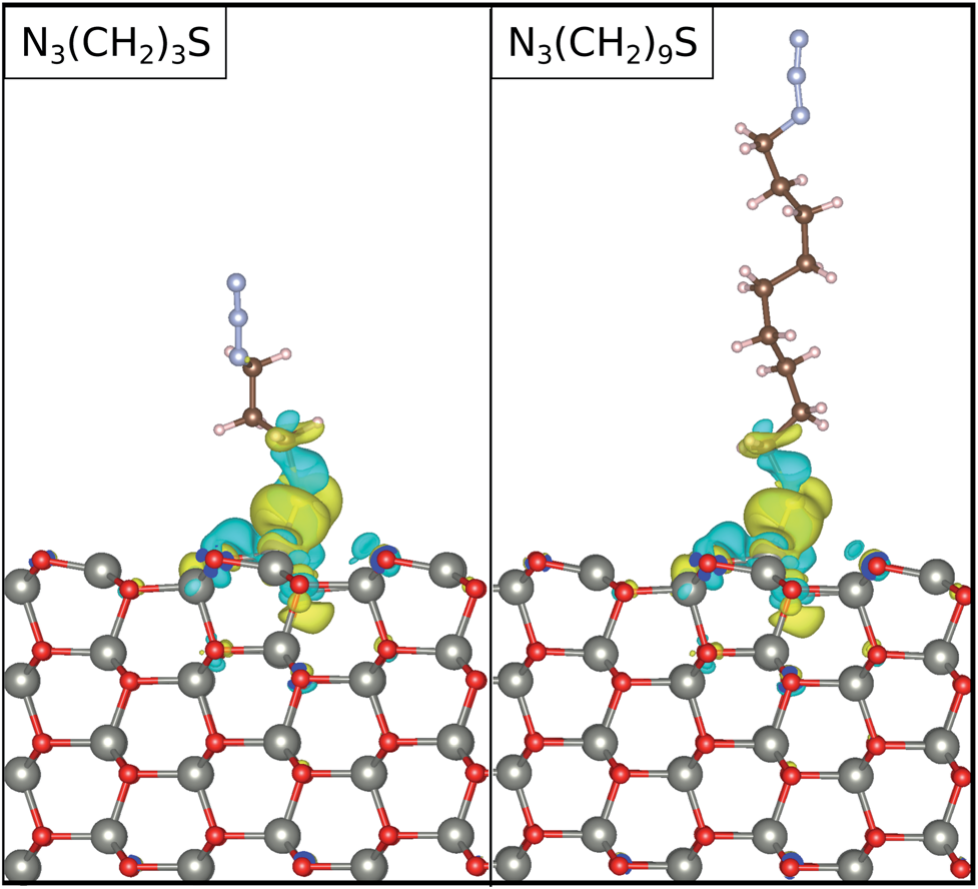
The electronic charge density difference for the functionalized surfaces. The results are shown for the molecular linker lengths of *n* = 3, and 9, as denoted in the legends. The blue and yellow colors denote charge accumulation and depletion, respectively. The isosurfaces are set at 0.001 electrons per Å^3^.

The trends in the charge accumulation on the adsorbed molecule are further supported by the electronic charge density difference. This difference, Δ*ρ*(*r*), for N_3_CH_2_S^−^ adsorbed on ZnO(101̄0) is defined through the expression:

5 Δ*ρ*(*r*) = *ρ*_tot_(*r*) − *ρ*_sub_ − *ρ*_ads_(*r*),

where *ρ*_tot_(*r*), *ρ*_ZnO_, and *ρ*_mol_(*r*) are the charge densities of the system of the N_3_CH_2_S^−^ adsorbed on ZnO(101̄0), the free standing ZnO(101̄0), and the free standing N_3_CH_2_S^−^, respectively. Note, that we have kept the atomic coordinates fixed with reference to the adsorbed configuration for calculating both *ρ*_ZnO_ and *ρ*_mol_(*r*). Upon adsorption, the charge densities on both N_3_CH_2_S^−^ and ZnO(101̄0) are rearranged with respect to their free standing counterparts. As depicted in Fig. 5, the electronic charge accumulates on the N_3_CH_2_S^−^ molecule, while it is depleted on the ZnO(101̄0) surface. The changes in the charge density emerge mainly on the N_3_CH_2_S^−^ and the surface O of the ZnO(101̄0). In addition, most of the charge density is being accumulated on the anchor site of the molecule and the lowest part of its alkyl chain, as well as the surface atoms nearest to the adsorption site. Accordingly, charge is moving from the surface towards the molecule, but remains localized at the interaction site. This results to a higher charge at the lower end of the SAM that could potentially be further moved towards the terminal group of the SAM with the application of an electric field possibly realizing photoelectric devices.

**Fig. 6:**
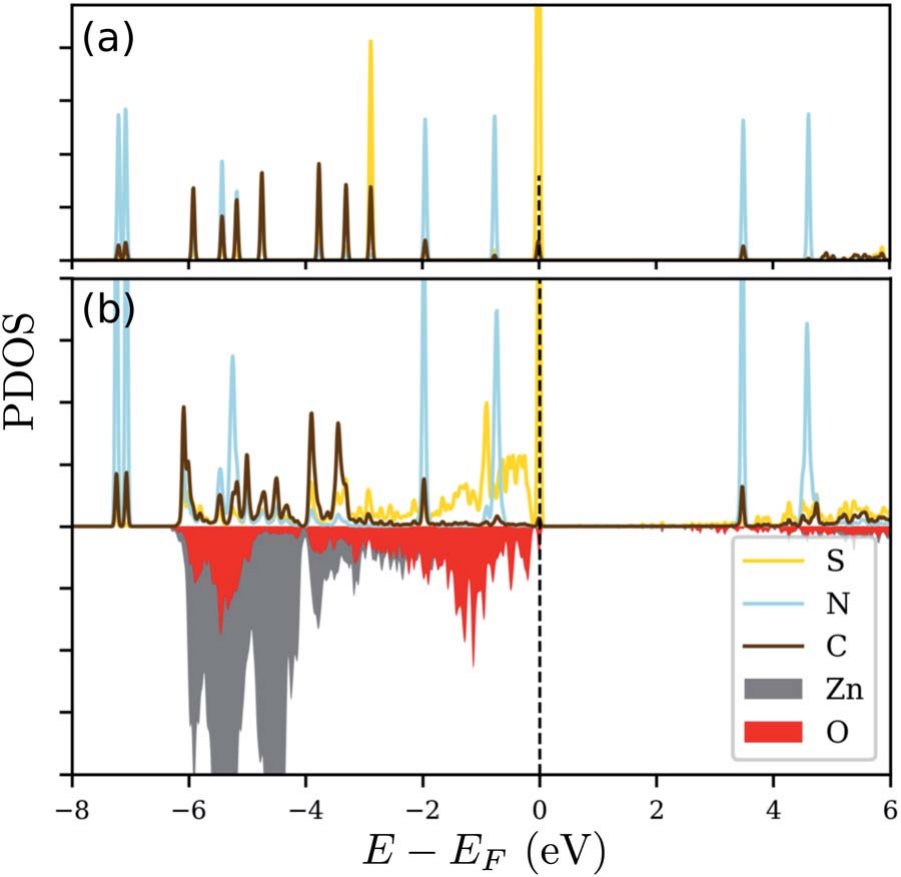
The projected density-of-states (PDOS) for (a) free standing N_3_(CH_2_)_3_S and (b) N_3_(CH_2_)_3_S adsorbed on ZnO(101̄0). The red and gray areas represent the contribution of the surface O and Zn atoms, respectively on the surface. The Fermi level (*E*_F_) is indicated by the dashed vertical line at 0 eV.

Since there is an excess charge of the molecule upon adsorption, we anticipate that the adsorbed molecule should play an important role in defining the electronic properties of the modified zinc oxide. In order to also reveal, whether the respective electronic states are indeed involved in the binding of N_3_CH_2_S^−^ on ZnO(101̄0), we show the projected electronic density of states (PDOS) in Fig. 6. Therein, the PDOS of the free standing N_3_CH_2_S^−^ and that of the adsorbed molecule on the zinc oxide surface are compared. The PDOS of the molecule is decomposed into the contributions from the different types of atoms of the head- and end-groups, S and N, as well as the ‘backbone’ atoms, C, of the alkyl chain. The surface is being considered through the contributions of the surface Zn and O atoms nearest to the molecule. Inspection of panel (a) in Fig. 6 reveals the very distinct peaks arising from degenerate S-, C- and N-based states. The electronic states of S and C lying below the Fermi level are being broadened after adsorption. The respective modification of the S states due to adsorption is very strong and is related to the charge accumulation on the adsorption site, as mentioned above. The case for the electronic states of nitrogen is different, as these are similar to those in the free-standing molecule. This can be expected also by the fact, that the charge analysis above did not reveal any important contribution of the azide terminal group. Based on this, the azide group does not play any role in the adsorption, nor changes its electronic signature.

Essentially, after adsorption of N_3_CH_2_S^−^, the orbitals of S below the Fermi level are hybridized with the orbitals of the surface oxygen and zinc atoms close to the sulfur, as can be inferred from the energy range of the broadening that is the same as the energy range of the S states. A strong interaction, thus modification of the electronic states of the free standing molecule, can be observed at energies corresponding to a higher contribution of the electronic states of Zn and O close to the adsorption site. Accordingly, the electronic states of the free standing molecule are still distinct and well defined outside this ‘interaction’ range. The same trends can be observed for the other molecules, as well. Quantitative differences can be found in the number of degenerate peaks (and their energies) of the isolated molecules and the energy window of the interaction between substrate and molecule upon adsorption.

### 3.2 Thin SAMs on the ZnO(101̄0)

**Fig. 7:**
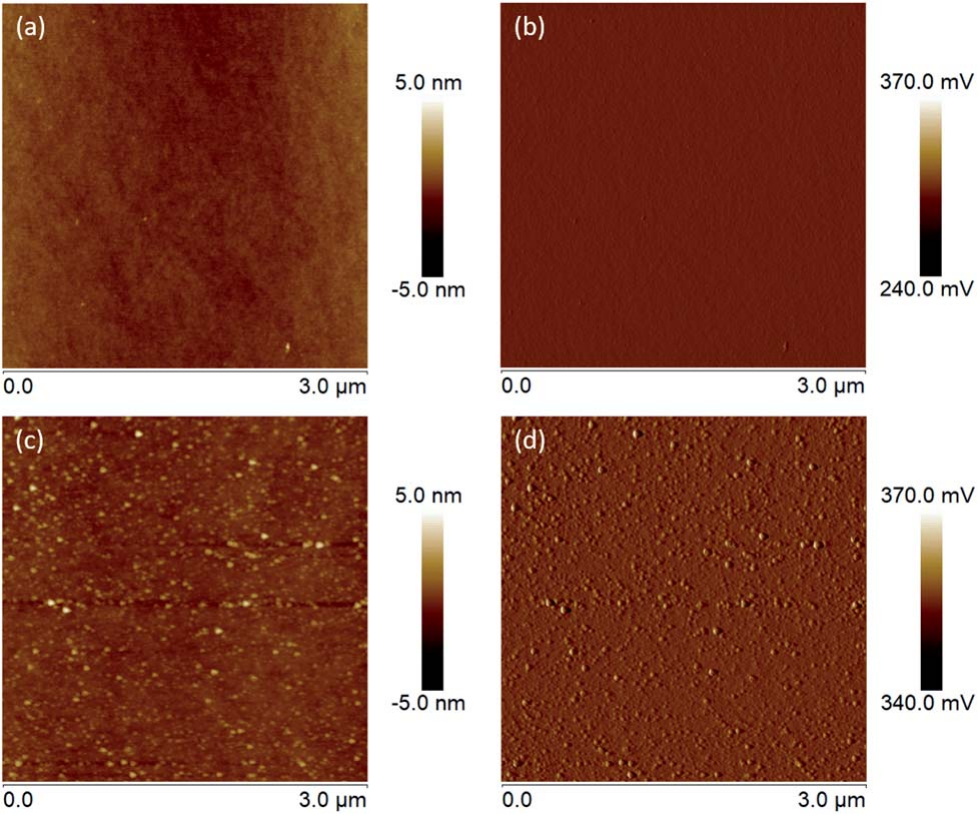
(a) Height and (b) amplitude AFM images of the clean ZnO(101̄0) surface. (c) Height and (d) amplitude AFM images of ZnO(101̄0) assembled with 3-azidopropyl thiol for 24 hours at room temperature.

From the intensively studied thiol-based chemistry, used to attach organic molecules on metal surfaces (Au, Ag, Pt, Pd and Hg), it is known that not only thiols but also disulfides and thioesters can be used as anchor groups for the SAM formation.^38,39^ However, compared with them thiol molecules form SAMs with the highest packing density. Therefore, since the affinity of thiols towards oxides is comparably lower than those towards metals, for our study 3-azidopropyl thiol was obtained from the corresponding thioacetate. The reaction was conducted under inert atmosphere to avoid the formation of disulfides. The removal of the acetyl group was confirmed *via*^1^H NMR with the disappearance of the signal at 2.35 ppm corresponding to the protons from the acetyl group (Fig. S1†). A complementary FTIR analysis of the obtained thiol confirmed the disappearance of the peak at 1685 cm^−1^ corresponding to the stretching vibrations of the carbonyl group, which was distinctly visible in the thioacetate FTIR spectrum, while the intensive peak at 2090 cm^−1^ assigned to the azide moiety remained in the thiol spectrum unchanged (Fig. S2†). The self-assembly of the 3-azidopropyl thiol on a ZnO(101̄0) surface was investigated *via* AFM. In Fig. 7, the height and amplitude AFM images of the ZnO substrate before and after the self-assembly are presented. As can be seen, the oxide surface becomes rougher after the assembly indicating the formation of an additional layer on the bare ZnO surface. However, the AFM analysis can only give a qualitative conformation for the deposition of an organic film on the oxide substrate. Since the thiols used in our experiments have a short alkyl chain, it is not possible to resolve a monolayer on the oxide surface using AFM. However, the increase in the surface roughness can be attributed to the formation of the SAM film.

**Fig. 8:**
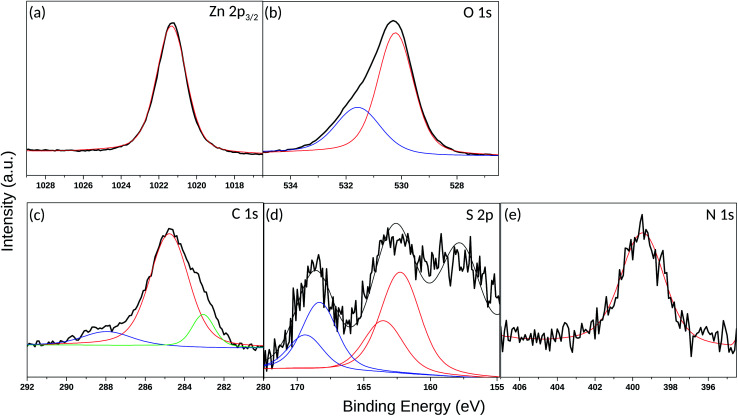
XPS spectra (black lines) and their Gaussian fits (colored lines) corresponding to the (a) Zn 2p_3/2_ peak, (b) O 1s peak, (c) C 1s peak, (d) S 2p peak and (e) N 1s peak for the ZnO (101̄0) surface assembled with 3-azidopropyl thiol molecules.

In order to gain deeper insights into the nature of the SAM layer on ZnO, XPS analysis was further performed. Temperature-dependent XPS was used to investigate the bonding to the oxide surface and the stability of the SAM film at elevated temperatures, while the SAM film thickness was determined *via* ARXPS. Fig. 8(a) reveals a Zn 2p_3/2_ single peak at 1021.2 eV, corresponding to the lattice zinc in accordance to previous studies.^72^ The O 1s spectrum in Fig. 8(b) consists of a two-component peak with a maximum at 530.3 eV, attributed to the lattice oxygen.^72^ The presence of a shoulder on the high-energy side of the O 1s peak, located at 1.3 eV above the main peak for the lattice oxygen (531.6 eV) could be assigned to –OH. The positions of the peaks are in a good agreement with those observed previously^72^ in the XPS O 1s photoelectron spectra for synthetic ZnO powder. Another possible explanation for the appearance of this shoulder could also be the presence of species with Zn–O–S bonding as also inferred elsewhere.^36^ The latter is very probable, considering that the self-assembly of the thiol molecules was performed immediately after cleaning of the ZnO(101̄0) substrate including as a final step, O_2_-plasma treatment.^73^ This treatment was required to remove residual contaminations and to make the surface more hydrophilic. Accordingly, the created reactive oxygen species on the substrate surface could interact chemically with the thiols and cause formation of a S–O bond. Fig. 8(c) shows an XPS narrow scan of the C 1s signal, which can be deconvoluted into three components. The most intensive peak with a maximum at 284.8 eV can be attributed to the carbon atoms involved in the alkyl chain of the thiol molecule.^74^ However, the asymmetric shape of the C 1s line (shoulder on the low-energy side) and the broadening of the peak prompts for the formation of thiol multilayers on the ZnO surface leading to differential surface charging and hence to a C 1s peak shift, as well as variations in the line-shape.^75^ The latter can also be supported by the AFM images (Fig. 7(c) and (d)) showing a rough and not homogeneous ZnO surface after the thiol assembly. Another possible reason for the C 1s peak broadening may also be due to the low number of carbon atoms in the alkyl chain and hence to a significant contribution of the C–N and C–S carbon atoms. According to the reported literature data, the binding energy (BE) peaks of carbon atoms next to electron-withdrawing elements such as nitrogen, sulfur and oxygen appear in the range 286–288 eV.^76–78^ Although the peaks for C–N and C–S carbon atoms are hidden in the tail of the main carbon peak, a weak additional peak appeared at 288 eV. It is associated with C=O carbon atoms, which might come from traces of the starting reactant (3-azidopropyl thioacetate), though, no residues of 3-azidopropyl thioacetate were visible on the ^1^H NMR spectrum of the synthesized thiol.

The sulfur 2p spectrum from the 3-azidopropyl thiol-SAM on ZnO substrate is shown in Fig. 8(d). The deconvolution of the S 2p line revealed the presence of three main components with peak maxima at 157.8 eV, 162.6 eV, and 168.6 eV. The peak at 157.8 eV is not considered for evaluation as it is attributed to a plasmon loss from the Zn 3s transition.^36^ The main S 2p peak at 162.6 eV corresponds to the S–Zn bond.^79^ Since the S 2p peak has closely spaced spin–orbit components (S 2p_3/2_ and S 2p_1/2_) with a difference of 1.16 eV, the fitting of the S 2p line here derived a BE of S 2p_3/2_ and S 2p_1/2_ at 162.3 eV and 163.6 eV, respectively. The appearance of this peak supports the shown in Fig. 3 predicted favorable molecule adsorption in site-4 and site-2. The BE for unbound thiols or disulfides at slightly higher values^74^ seems to be hidden in the peak tail. The third peak in the S 2p spectrum centered at 168.6 eV is assigned to S–O bonding, which can be explained either with oxidation of the adsorbed thiol molecules or SAM molecules with the oxygen sublattice resulting in S–O–Zn bonding as already discussed above for the O 1s spectrum (Fig. 8(b)). Finally, a single peak in the N 1s spectrum (Fig. 8(e)) appeared at 399.5 eV and could confirm the presence of the azide terminal group in the SAM molecule.

**Fig. 9:**
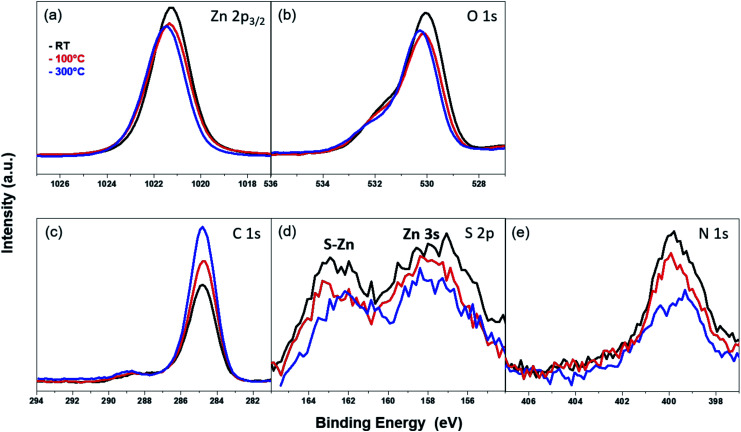
XPS spectra of the (a) Zn 2p_3/2_ peak, (b) O 1s peak, (c) C 1s peak, (d) S 2p peak and (e) N 1s peak for the ZnO (101̄0) surface assembled with 3-azidopropyl thiol molecules annealed to different temperatures (room temperature ‘RT’ (black curve), 100 °C (red curve) and 300 °C (blue curve)) in UHV.

The thermal stability of the azide-functionalized linkers attached to the ZnO surface was examined *via* temperature-dependent XPS. The corresponding XPS spectra were recorded at RT after annealing at 100 °C and 300 °C in UHV. The results for the Zn 2p_3/2_ peak at different temperatures are given in Fig. 9(a). The intensity of the peak reduces slightly at 100 °C, but does not change significantly with a subsequent rise in temperature. The position of the peak shifts by 0.1 eV towards higher binding energies after annealing. A similar trend is observed with the O 1s peak (Fig. 9(b)), though, in addition, the intensity of the shoulder above the lattice oxygen peak also decreases with an increase in temperature. However, the C 1s peaks (Fig. 9(c)), show an opposing trend in the temperature-dependent XPS spectra. The intensity of the peaks increases with an increase in temperature. This can be correlated with an increase in the film thickness explained in the coming section. The absence of additional peaks which often appear as a result of differential charging could be attributed to the absence of multilayers.^80^

The S 2p and N 1s peaks (Fig. 9(d) and (e)) corresponding to the S–Zn bond and azide terminal group, respectively, show similar behavior with increasing temperature. At 100 °C, the intensity of both peaks starts to decrease, and the effect becomes pronounced at 300 °C, accompanied additionally with a shift of the peak towards lower BE. This shift in the peak positions could be indicative of chemical changes in the organic molecule. From these temperature-dependent XPS measurements, it can be posited that the thiol-containing SAMs have relatively poor thermal stabilities. After heating at 100 °C, a marked decrease in their chemical integrity can be observed. This could be a sign of decomposition, chemical transformation or desorption of the SAM at higher temperatures. Our observations with the azide-functionalized thiol SAM on ZnO (101̄0) surface are in good agreement with the results found for the thermal stability of similarly-sized but unfunctionalized *n*-hexanethiol assembled on ZnO film sputter-grown on molybdenum foil.^36^

In order to confirm whether the azide-functionalized linkers build a mono- or multilayer on the ZnO surface, the SAM film thickness was calculated *via* ARXPS from the attenuation of the Zn 2p_3/2_ photoelectron line (see the experimental part). At room temperature, a film thickness of 3.03 + 0.03 Å was obtained. Since this value is lower than the length of the SAM molecule (8.7 Å) – obtained by structure modeling (ACD/3D optimizing algorithm^81^), the SAM film on the ZnO surface can be considered to form a monolayer. This is in good correlation with the results from the temperature-dependent XPS analysis of the C 1s region. The measured thickness is comparable with the film thickness of 3.3 ± 0.4 Å measured for the similarly-sized but *n*-hexanethiol SAMs assembled on ZnO for 16 hours.^36^ The film thickness of the azide-functionalized SAM was also measured after heating the sample at 100 °C and 300 °C, which yielded 3.17 + 0.05 Å (after 100 °C treatment) and 3.57 + 0.05 Å (after 300 °C treatment). The slight increase of the film thickness could be explained with an enhanced molecule mobility on the ZnO surface at higher temperature. This might cause changes in the conformation and the molecules stretch further due to the heat treatment.

The BE of the ZnO(101̄0), N_3_(CH_2_)_3_S, and N_3_(CH_2_)_3_S adsorbed on ZnO(101̄0) are calculated using the initial state approximation and is presented in Table 3. Despite limitations of the initial state approximation and the differences between the synthesised SAMs and the simulated structures, the calculated BEs capture the trends of the experimental measured ones (Table 3 and Fig. 8). The 2p core level BE of the surface Zn (998.01 eV) bonded with a thiol molecule is about 1.43 eV smaller than that of the surface Zn (999.44 eV). The negative ΔBE relates to changes in the coordination of the surface Zn atom. Upon adsorption of the thiol molecule, the orbitals of the S atom hybridize with the orbitals of the Zn atoms. This hybridization gives rise to a re-distribution of the valence-level electrons (6), thus changing the electrostatic potential at the nucleus of the surface Zn and O atoms. Consequently, an upward shift (negative ΔBE) of the core-level orbital-energy occurs.

**Table 3:** Summary of the calculated BEs and ΔBEs for the ZnO(101̄0), N_3_(CH_2_)_3_S, and N_3_(CH_2_)_3_S adsorbed on ZnO(101̄0) (N_3_(CH_2_)_3_S@ZnO(101̄0)) using the initial state approximation. The values obtained correspond to ground state orbital energies. All the energies are shown in eV

Parameters	Orbitals	BE	ΔBE
N_3_(CH_2_)_3_S	S(2p)	149.34	0.00
N_3_(CH_2_)_3_S	C(1s)	264.66	0.00
N_3_(CH_2_)_3_S	N(1s)	376.88	0.00
ZnO(101̄0)	Zn(2p)	999.44	0.00
ZnO(101̄0)	O(1s)	504.62	0.00
N_3_(CH_2_)_3_S@ZnO(101̄0)	S(2p)	149.45	0.11
N_3_(CH_2_)_3_S@ZnO(101̄0)	C(1s)	264.81	0.15
N_3_(CH_2_)_3_S@ZnO(101̄0)	N(1s)	377.15	0.27
N_3_(CH_2_)_3_S@ZnO(101̄0)	Zn(2p)	998.01	−1.43
N_3_(CH_2_)_3_S@ZnO(101̄0)	O(1s)	504.20	−0.42

## 4 Conclusions

Using computer simulations, we have modelled the adsorption of N_3_(CH_2_)_*n*_SH molecules with lengths *n* = 1, 3, 6, 9 on a ZnO(101̄0) surface. A SAM formation with one of these molecules namely 3-azidopropyl thiol N_3_(CH_2_)_3_SH on ZnO (101̄0) surface was also experimentally confirmed. We have further analyzed the structural and energetic components of these modified substrates. The molecules were found to prefer binding on the bridge side to the two neighboring surface Zn atoms *via* two Zn–S bonds. Increasing the length of the carbon chain gives rise to a stronger adsorption (larger adsorption energy). We have found a linear correlation of the distortion energy of the ZnO(101̄0) surface and its interaction energy with the molecule. A strong accumulation of the electron density on the adsorbed molecule was evident, underlying the shift of the charge towards the molecule due to adsorption. The electronic calculations revealed that the atomic orbitals of the free standing molecules do hybridize with those of the surface atoms giving an additional evidence of the chemisorption.

The results from the computer simulations were additionally supported by the conclusions drawn from the XPS analysis of the experimentally assembled azido-functionalized thiol molecules on the ZnO surface. The main peak in the sulfur 2p spectrum at 162.6 eV, assigned to the S–Zn bound, confirmed a direct interaction of the thiol group of the linker molecules and the substrate. The latter is in a good agreement with the predicted favorable molecule adsorption in site-4 and site-2 (Fig. 3). Considering the significantly higher and not practically achievable energies for adsorption sites with oxygen contribution, such as site-1 and site-5, one can conclude that the peak at 168.6 eV in the sulfur 2p spectrum, assigned to S–O bonding, appeared rather due to oxidation of the adsorbed thiol molecules than due to their interaction with the oxygen sublattice. The reduction in the intensities of the S 2p and N 1s peaks and shift of their respective peaks towards lower BEs with increasing the temperature revealed that the 3-azidopropyl thiol SAM starts to degrade around 100 °C. ARXPS measurements show that a monolayer is formed on the ZnO substrate with a thickness of 3.03 + 0.03 Å at room temperature. This is in a good agreement with the film thicknesses reported for unfunctionalized thiols with comparable chain lengths. The increase in the SAM film thickness at higher temperatures (3.17 + 0.05 Å (after 100 °C treatment) and 3.57 + 0.05 Å (after 300 °C treatment)) can be attributed to the increased mobility of the SAMs at higher temperatures.

Our work is a proof-of-principles study on the adsorption of small organic molecules through a thiol group on oxide surfaces underlying the essential characteristics that can be tuned in order to stabilize the adsorption. Further investigation should focus on the influence of other hybrid features, such as the surface coverage and the effect of different anchor groups on the stability of the functionalized metal oxides. Further, the influence of environmental factors, such as the presence of a solvent, possible strain, *etc.* needs also to be evaluated. For such investigations, our work on the adsorption of single small organic molecules on a metal oxide surface and the SAM formation serve as a basis. As a general remark, the organic–inorganic hybrid materials studied here can be further utilized as biomedical templates or functionalized porous matrices for heterogeneous catalysis. For these, the materials' properties can be tuned through the SAM composition in the hybrid. The novelty of our work is directed mainly on providing templates for further functionalization using ‘click’ chemistry, thus enhancing the functionality of the hybrid materials. The selective choice of the functional end-group of the SAMs can further tune the properties of the material and provide selectivity towards specific ‘click’ molecules. In this way, a broad spectrum of functional hybrids can be synthesized depending on the desired functionality and application in mind.

## Conflicts of interest

There are no conflicts to declare.

## Supplementary Material

RA-011-D0RA05127F-s001
