# Supplementary material for: Adsorption of azide-functionalized thiol linkers on zinc oxide surfaces
Source: RSC Adv. 2021 Jan 29;11(10):5466–78. doi: 10.1039/d0ra05127f (PMC8694732; doi:10.1039/d0ra05127f)
Supplement: RA-011-D0RA05127F-s001 [file RA-011-D0RA05127F-s001.pdf]

Electronic Supplementary Material (ESI) for RSC Advances.
This journal is © The Royal Society of Chemistry 2021

Supplementary Information

Adsorption of azide-functionalized thiol linkers on zinc oxide surfaces

Petia Atanasova[1], Maofeng Dou[2], Shravan R. Kousik[1], Joachim Bill[1], and Maria Fyta[2]

[1]*Institute for Materials Science, University of Stuttgart, Heisenbergstr. 3 70569 Stuttgart, Germany*

[2]*Institute for Computational Physics, University of Stuttgart, Allmandring 3, 70569 Stuttgart, Germany*

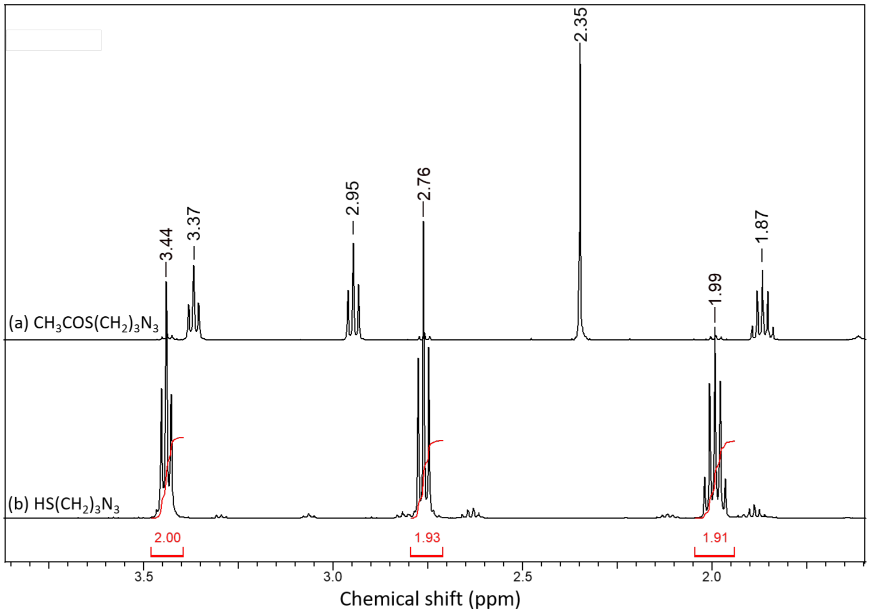

Figure 1: ^{1}H NMR spectra of (a) S-(3-azidopropyl) thioacetate and (b) 3-azidopropyl thiol.

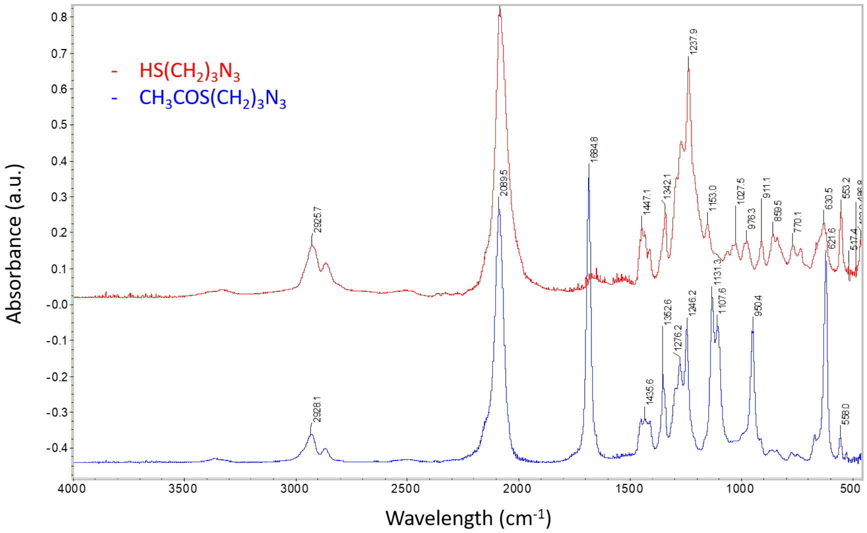

Figure 2: FTIR-ATR spectra of S-(3-azidopropyl) thioacetate (blue curve) and 3-azidopropyl thiol (red curve).
